# Initial investigation of kinesiophobia as a predictor of functional reaction time one year after concussion

**DOI:** 10.2217/cnc-2023-0014

**Published:** 2024-05-07

**Authors:** Melissa N Anderson, Robert C Lynall, Patrick J O'Connor, Julianne D Schmidt

**Affiliations:** 1 Ohio Musculoskeletal & Neurological Institute, Ohio University, Athens, OH 45701, USA; 2 College of Health Sciences & Professions, Ohio University, Athens, OH 45701, USA; 3 UGA Concussion Research Laboratory, Department of Kinesiology, University of Georgia, Athens, GA 30605, USA; 4 Exercise Psychology Laboratory, Department of Kinesiology, University of Georgia, Athens, GA 30605, USA

**Keywords:** college students, dual-task cost, fear, mild traumatic brain injury, response time

## Abstract

**Aim:**

The relationship between post-concussion kinesiophobia and clinical and functional reaction time (RT) beyond clinical recovery remains to be elucidated.

**Methods:**

College-aged participants with (n = 20) and without (n = 20) a concussion history completed patient-reported outcomes, and RT tasks. Kinesiophobia, symptoms and RTs were compared using t-tests. Linear regressions were performed to determine if kinesiophobia predicted RT measures and dual-task cost.

**Results:**

The concussion history group reported higher scores (p < 0.01) for all patient-reported outcomes. We observed significant single-task RT differences between groups (p = 0.013) such that those without a concussion history (m = 0.51s ± 0.08) were faster (m = 0.59s ± 0.12). There were no clinical or dual-task RT differences between groups (p > 0.05). Kinesiophobia significantly predicted single-task RT (R^2^ = 0.22).

**Discussion:**

Kinesiophobia should be considered when measuring RT.

Concussions are complex injuries resulting in a myriad of signs and symptoms, such as headache, nausea, dizziness, difficulty concentrating, and emotional disturbances [1]. They can also lead to deficits in motor function, including balance, gait, coordination and reaction time (RT). Post-concussion RT deficits are widely recognized as an important indicator of impaired brain function and have been reported to extend beyond the traditional clinical recovery timeline of 28 days [2]. Reaction time is a critical component of sports performance and activities of daily living and may play a role in subsequent musculoskeletal injury risk, which remains doubled for up to 2 years after a concussion [3–7]. The underlying mechanism for this increased injury risk remains to be elucidated, as no clinical predictors (i.e., Standardized Assessment of Concussion, Balance Error Scoring System, Computerized Neurocognitive testing, clinical reaction time, demographic or injury characteristics) have been found to identify subsequent injury risk [8]. Further research is needed to identify why certain people sustain a subsequent injury as well as explore the potential role of psychological factors in influencing the risk of post-concussion injuries.

Despite the recent calls for research on the emotional consequences of sport-related concussions [9], there is a limited understanding of the psychological factors that may affect recovery and subsequent injury risk. One such factor that is often overlooked is kinesiophobia, which is characterized by a fear of movement and physical activity, stemming from a sense of vulnerability caused by injury and is frequently accompanied by anxiety [10,11]. Kinesiophobia is well-studied across other sports injuries and has been reported to significantly increase the risk of musculoskeletal injury, specifically anterior cruciate ligament tears [10,12], yet this topic has received limited attention following concussion [13,14]. A recent study on adolescents with concussions reported high kinesiophobia is related to vestibular/ocular motor symptom provocation [14], with another study finding a significant relationship between kinesiophobia and clinical reaction time at medical clearance such that greater kinesiophobia resulted in slower reaction time [13]. While evidence suggests a relationship between maladaptive mood states (e.g., anxiety, depression) [13] and clinical reaction time following concussion, there is a paucity of research on kinesiophobia related to functional RT. Clinical RT testing, usually accomplished through computerized neurocognitive testing, has long been considered an important component of a multifaceted concussion care and management approach. However, a major limitation of clinical RT is that it is not correlated to sport-like functional RT [15]; thus, it may not apply to actual subsequent injury risk or neurological recovery from concussion.

Therefore, the purpose of this exploratory study was to describe and compare kinesiophobia between groups with and without a recent history of concussion and determine if it was correlated with perceived recovery and determine if kinesiophobia was significantly associated with clinical RT, functional RT, and dual-task cost in the concussion history group. By examining the relationship between these factors, we aimed to enhance our understanding of the extent to which psychological factors such as fear of injury are associated with reaction time and subsequent injury risk. We hypothesize that higher levels of kinesiophobia will be significantly associated with lower perceived recovery and higher (i.e., worse) RTs and dual-task cost [16].

## Materials & methods

No sample size estimation was performed for this exploratory analysis and a convenience sample of 20 concussion history and 20 control participants were enrolled (Table 1). Non-varsity athlete college-aged students with a concussion history were recruited from a patient database at a university health center and controls from the university student body between December 2020 and August 2021. Due to the COVID-19 pandemic, a five-month recruitment hiatus occurred. Consequently, to address the study's primary objective of investigating the relationship between persistent kinesiophobia and reaction time measures, we opted to invite potential participants one-year post-concussion. This decision is further supported by existing literature showing that there is an increased risk of musculoskeletal injuries up to 2 years following a concussion [3,4,17,18]. Concussion history participants were included if they had a medically diagnosed concussion in the past 12 ± 2 months (m = 11.8 ± 1.7) and had previously consented to be contacted for research purposes. They were excluded if they self-reported signs of a more severe brain injury (e.g., positive imaging) or reported feeling less than 80% recovered from their most recent concussion. In addition, all participants were excluded if they had a history of lower-extremity musculoskeletal injury <180 days that resulted in >24 hours of time loss from activity, reported a history of 3+ prior concussions, reported low levels of physical activity (<75 min per week of at least moderate exercise), or had a neurological disorder (i.e., migraines, ADHD). Further, potential control participants were excluded if they reported a lifetime concussion history. All participants provided written informed consent prior to study participation. The University of Georgia Institutional Review Board approval was obtained prior to data collection (PROJECT00002606) and signed informed consent was obtained from all participants.

**Table 1.** Mean (SD) demographic Information and p-values for the groups.

	Concussion history	Control	p-value
Age in years (SD)	20.25 (1.37)	20.65 (1.27)	0.845
Height (cm) (SD)	172.87 (9.10)	173.98 (8.29)	0.979
Mass (kg) (SD)	67.91 (14.06)	69.52 (9.14)	0.115
Years of education (SD)	14.85 (1.31)	14.95 (1.10)	0.422
Sex^†^			0.002
Male (n; %)	4 (20%)	10 (50%)	
Female (n; %)	16 (80%)	10 (50%)	
Lifetime history of concussion			
Yes	20 (100%)	0 (0%)	-
More than one prior concussion			
Yes	1 (5%)	0 (0%)	-

† Chi-square indicated a significant difference between the groups at the p < 0.05 level.

### Patient-reported outcome measures

#### Self-reported recovery

Participants in the concussion history group self-reported how recovered they felt from their most recent concussion on a visual analog scale ranging from 0% (not at all recovered) to 100% (completely recovered).

#### SCAT5 symptom inventory

Symptoms were assessed using a 22-item symptom checklist from the Sport Concussion Assessment Tool (SCAT5) [19]. Participants reported the severity of each symptom on a seven-point Likert scale (0 = “not at all”; 6 = “severe”). Total symptoms (range 0–22) and symptom severity (range 0–132) were calculated.

#### Tampa Scale of Kinesiophobia

The Tampa Scale for Kinesiophobia (TSK) is a 17-item self-report, 4-point Likert scale (1 = “strongly disagree” 4 = “strongly agree”) that assesses kinesiophobia. Kinesiophobia is thought to involve excessive and debilitating fear of physical movement and activity resulting from a feeling of vulnerability to painful injury or re-injury [20]. The TSK was originally developed to measure the fear of movement with respect to low back pain and evidence supports the reliability and validity of the scores in a variety of samples [21]. TSK scores range from 17 to 68, whereas a higher score indicates a higher degree of kinesiophobia, and a score ≥ 37 was considered high [22]. Consistent with prior research [14], we used a modified version for our concussion history group such that items were specific to their most recent concussion. Control participants were asked to respond to each question in the general context of any injury.

### Reaction time tasks

#### Clinical reaction time task

The Stroop Task test was completed using a validated and reliable computerized neurocognitive test measure (CNS Vital Signs, LLC, Chapel Hill, NC). The Stroop effect has been described in detail elsewhere [23] but briefly uses cognitive interference to generate simple RT (participant responds as quickly as possible to a word appearing on the screen), complex RT (participant responds as quickly as possible when the word on the screen matches the color font), and Stroop RT (participant responds as quickly as possible when the word on the screen does not match the color font) in seconds (s). All clinical RTs were calculated as the average time in seconds between stimuli presentation and when the participant pressed the spacebar on the computer keyboard. These three scores were then weighed together to make a composite RT widely used in clinical practice.

#### Functional reaction time task

**Figure 1.**
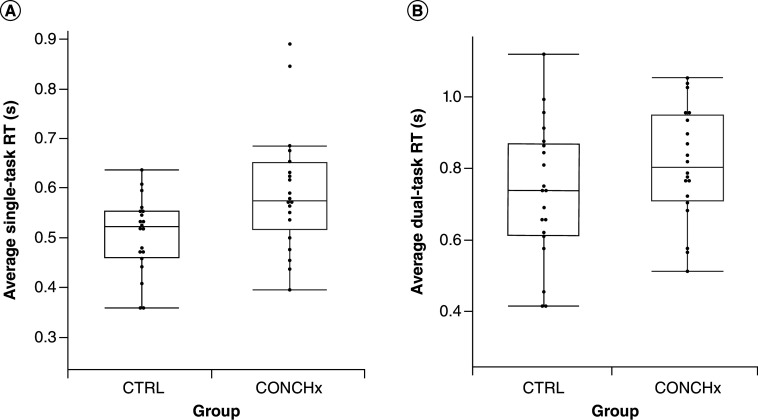
Box plots of functional reaction time. **(A)** Single-task (p = 0.013) and, **(B)** dual-task (p = 0.172) conditions between groups. RT: Reaction time.

Our functional RT task was jump landing assessments that occurred in an eight-camera 3D motion capture space (MIQUIS; Qualisys Systems, Goteborg, Sweden), recording at 240 Hz. Participants were fitted with a passive reflective marker cluster over their posterior superior iliac spines and sacral body (three markers total). Participants stood on a 30 cm box at 50% of their height away from a target awaiting a visual stimulus which was a green light 3 m in front of them [15,24]. Participants took an athletic stance to anticipate the stimulus, responding by jumping onto the target as quickly as possible and immediately performing a maximum vertical leap (16 trials: eight single-task, eight dual-task) (Figure 1). Participants were required to complete two familiarization trials before data collection began. Functional RTs were calculated by the time (s) from visual stimulus onset to the participant's first movement. The first movement was defined as ≥3 cm of movement of the sacrum marker in either the sagittal or transverse plane [15,24].

Reaction time assessments were completed under both single-task (i.e., *“Please focus solely on completing the functional task as quickly as possible when you see the visual stimulus”*) and dual-task (i.e., *“Please subtract as quickly and accurately as possible as you await your visual stimulus. Once the visual stimulus occurs, I want you to complete your functional task as quickly as possible”*) conditions. All the instructions gave equal weight to speed and accuracy. The dual-task instructions gave equal importance to the functional and cognitive tasks. The cognitive task was subtracting aloud from a random number between 99 and 299 by either 6's or 7's. A new number was introduced before each trial.

Finally, dual-task cost ([dual-task RT – single-task RT/single-task RT]*100) was calculated for functional tasks. Dual-task costs quantify the amount of change between undivided and divided attention and can distinguish between concussed and control patients [7,25–27] thus an important metric of performance.

### Data processing

Sacral marker positional data were imported to Visual 3D (Visual 3D, C-Motion Inc., MD, USA). Data were processed and filtered using a 4th order, low-pass Butterworth filter with a 10 Hz cutoff frequency, and trials were averaged to produce mean reaction time scores for each assessment.

### Statistical analysis

Independent t-tests compared groups on total symptoms, symptom severity, TSK scores, functional and clinical RTs and dual-task cost. Effect sizes were calculated using Cohen's d (small = 0.2, medium = 0.5, large = 0.8) [28] and 95% confidence intervals (CIs) were provided for all group comparisons. A Pearson correlation was conducted to determine if kinesiophobia was significantly related to perceived recovery.

Four separate linear regressions were performed to determine if kinesiophobia predicted clinical RT, functional (i.e., single-task and dual-task) RT, and dual-task cost. Models included the predictor variables group*kinesiophobia, group, and kinesiophobia. We chose not to include sex as a predictor because we found no significant differences in RT between males of females for any of the tasks or conditions (p range = 0.166–0.059) during preliminary analysis. We applied the Benjamini–Hochberg correction to control the false discovery rate in multiple hypothesis testing [29,30]. While not as aggressive as a familywise correction (i.e., Bonferroni), this method was selected due to the exploratory nature of this study. The newly calculated p-values ranged from 0.306 to 0.027. Thus, we applied a q-value of 0.025 to all analyses. All data, analysis code, and research materials are available upon request to the corresponding author. All analyses used JMP version 16.0 (JMP Statistical Discovery LLC).

## Results

Descriptive statistics and frequencies are presented in Table 2.

**Table 2.** Comparisons of patient reported outcomes and reaction times between groups.

Patient reported outcomes	Concussion History M ± SD	Control M ± SD	p-value	Cohen's d
Total symptoms^†^	5.3 ± 5.0	2.4 ± 0.5	0.009	0.82
Symptom severity^†^	10.0 ± 12.9	3.1 ± 4.5	0.017	0.71
Tampa scale of kinesiophobia total score^†^	41.3 ± 7.1	35.4 ± 6.3	0.004	0.87
	n (%)	n (%)		
Tampa scale of kinesiophobia – high kinesiophobia classification^†^	15 (75%)	7 (35%)	0.011	

Reaction time	M ± SD (95% CI)	M ± SD (95% CI)	p-value	Cohen's d
Clinical RT (s)	0.97 ± 0.18 (0.88–1.05)	0.95 ± 0.12 (0.89–1.00)	0.362	0.13
Single-task RT (s)^†^	0.59 ± 0.12 (0.53–0.64)	0.51 ± 0.08 (0.47–0.54)	0.013	0.78
Dual-task RT (s)	0.81 ± 0.15 (0.74–0.88)	0.73 ± 0.19 (0.64–0.82)	0.086	0.46
Dual-task Cost (%)	41.77 ± 35.06 (24.77–57.58)	44.19 ± 28.58 (30.81–57.56)	0.610	0.07

† Indicates significant differences between the groups at the p < 0.05 level.

SD: Standard deviation.

### Comparisons of patient-reported outcomes

The concussion history reported higher (i.e., worse) total symptoms (p = 0.009), symptom severity (p = 0.017), and TSK total scores (p = 0.004) than the control group (Table 2).

### Reaction time comparisons

We observed significant differences and a large effect size (Cohen's d = 0.78) in single-task RT between groups (p = 0.013) such that controls (M = 0.51 ± 0.08 seconds) were faster than the concussion history group (M = 0.59 ± 0.12 seconds) (Table 1). There were no group differences for clinical RT (p = 0.306) or dual-task RT (p = 0.086) between the concussion history and control groups (Table 2).

### Correlations

Kinesiophobia was not significantly correlated with perceived concussion recovery (r(18) = -0.179, p = 0.463).

### Regression models

Kinesiophobia (b = 0.415, p = 0.014) significantly predicted single-task RT (R^2^ = 0.22, p = 0.025) such that those with higher kinesiophobia has worse RT. No other predictors were significant in the model (group p = 0.035, group*kinesiophobia p = 0.129). Our models did not explain a significant proportion of the variance in clinical RT (R^2^ = 0.06, p = 0.451), dual-task RT (R^2^ = 0.20, p = 0.045) or dual-task cost (R^2^ = 0.05, p = 0.585).

## Discussion

Due to the unknown effects of kinesiophobia on functional RT, we described and compared TSK scores and RTs between college students with and without a concussion history. The primary finding of this study is that group and kinesiophobia predicted approximately 20% of the variance in single-task RT, such that having a recent concussion history and reporting movement avoidance resulted in significantly slower functional RT. This was not present for the clinical RT assessment, which occurs in a controlled environment while the participant is seated in front of a computer. Fear of injury appears to be an important moderator for functional RT and should be considered in future study designs, especially when investigating subsequent injury risk. Further, this investigation identified differences in RT and kinesiophobia between nonathletes with a concussion 1 year prior to testing and a healthy control sample, thus providing results that may be more generalizable to the nonathlete population.

Findings from this study suggests that fear of movement may be a long-term consequence of a concussion, extending beyond the acute recovery phase, as we observed significantly higher kinesiophobia ratings in our concussion history group. The personal experience of a concussion, coupled with its potential consequences, such as a greater perceived risk of injury, could lead to heightened levels of anxiety and a reluctance to be physically active [31]. The higher level of kinesiophobia within the concussion group underscores the potential importance of addressing and managing fear of movement in individuals who have a history of concussion in order to promote their overall well-being and functional recovery. Further, 75% of our concussion history group reported kinesiophobia above clinical cutoffs [32] while only 35% of the control group did. Clinicians could identify those with elevated kinesiophobia after a concussion and educate individuals to understand how movement avoidance could be contributing to post-concussion symptoms and related concerns [33].

The concussion history group had significantly higher total symptoms and symptom severity despite reporting feeling at least 80% recovered from their concussion. Existing literature suggests that a substantial minority of people continue to report concussion symptoms at 3 [34–37], 6 [38] and 12 [39] months post-concussion. While persistent symptoms are not uncommon, this is complicated because many concussion symptoms are non-specific and often reported in healthy adults. Considering the nonexclusivity of concussion symptoms, our concussion history group still reported significantly more symptoms than the control group. However, it is essential to note that this finding for the single task can largely be attributed to two participants in the concussion group who reported 47 and 39 symptoms, respectively, despite self-reporting being recovered and being medically cleared by their managing clinician. Furthermore, our findings did not suggest a significant relationship between self-reported recovery and kinesiophobia. Rather, fear of injury appeared to operate independently of perceived recovery.

Large reaction time deficits are recognized indicators of impaired brain function and have been reported to extend well beyond the ‘traditional’ concussion recovery timeline of 28 days, potentially increasing subsequent injury risk [2]. Despite our analyses identifying significant differences between the groups for functional single-task RT, there were no group differences in clinical RT. The Stroop Task test we used is a common and reliable RT assessment used within computerized neurocognitive testing however, it is not correlated with functional RT [15]. Our findings, in conjunction with previous work, further highlights the inability of current clinical assessments to detect lingering RT deficits after a concussion [15,40,41].

When examining the single-task reaction time, it was noteworthy that the concussion history group demonstrated a significantly slower RT compared with the control group, suggesting that even a year after the injury, individuals with a history of concussion experience lingering effects on their cognitive processing speed. Although small differences, these findings provide further evidence that concussion-related cognitive impairments may persist well beyond clinical recovery [2] and have implications for daily activities requiring multitasking and decision-making. Further research is warranted to explore the underlying mechanisms and potential interventions to support individuals with concussion history in their cognitive recovery.

As expected based on prior research, both groups exhibiting longer RTs under the dual-task compared with the single-task condition. The nonsignificant differences in dual-task cost between the groups is a novel observation. Dual-task costs are measures that represent the amount of change between undivided and divided attention. These costs can distinguish between concussed and control patients [25] as well as predict prolonged recovery [42], and are associated with time-loss injuries up to a year after a concussion [26]. The null findings here may be due to the nature of our dual-task condition, such that it was not complex enough to elicit significant between-group differences. Future research should consider using other functional tasks (i.e., walking, cutting drills) in the context of recent concussion history and fear of injury.

## Limitations

A limitation of this study is that there was no equal representation of males and females in the concussion history group. Most participants in the control group were tested in the early months of the investigation compared with most of the concussion participants. This potential confound is discounted by the weak evidence that human cognition varies by season [43]. Equal sex representation in the control group was based on historical patient records that showed a similar number of males and females seeking care for their concussion. Recruitment for participants in the concussion group occurred over 10 months and resulted in the enrollment of a significantly greater number of females than males (p = 0.002). While some studies have reported males to have significantly faster RT than females [44–46], others have found no differences between the sexes [47–50]. Participants in the concussion group were almost a year removed from their injury. This large time interval from concussion to assessment was primarily due to the COVID-19 pandemic but was chosen because of the proximity to subsequent injury occurrence. Future work should look at a time point closer to medical clearance to return to play to learn more about how kinesiophobia and RT recover at this important clinical time point.

## Conclusion

Kinesiophobia scores accounted for a substantial portion of the variance in dual-task RT, resulting in significantly slower RT due to fear of injury. Furthermore, the persistence of symptoms in the concussion group, even when self-reported recovery is high, highlights the complex nature of concussion symptoms and the need for improved assessment tools. Reaction time deficits are indicators of impaired brain function that extend beyond the traditional recovery timeline, emphasizing the necessity for better clinical assessments to detect lingering cognitive impairments. Individuals with a history of concussion experience slower cognitive processing speed even 1 year after the injury, highlighting the long-term effects on daily activities requiring multitasking and decision making. Future research should explore underlying mechanisms and interventions to support cognitive recovery in individuals with a concussion history.

**Summary points**

- Individuals with a concussion history may experience lingering reaction time (RT) deficits, including slower functional RTs, even after surpassing the traditional 28-day recovery window. This highlights the potential for increased subsequent injury risk.
- College students with a history of concussion exhibit slower functional RT and higher levels of kinesiophobia (fear of movement) compared with those without a concussion history.
- Fear of movement appears to be a significant factor influencing functional RT and may persist even after a person feels recovered from a concussion.
- Addressing kinesiophobia is crucial in post-concussion recovery to promote overall well-being and functional recovery.
- Kinesiophobia may be a long-term consequence of concussion, as evidenced by significantly higher scores in individuals with a concussion history.
- Individuals with a concussion history reported higher symptom severity and total symptoms compared with controls, even when reporting feeling recovered.
- The relationship between kinesiophobia and perceived recovery appears independent.
- More complex dual-task assessments might be needed to reveal differences in dual-task costs between individuals with and without concussion history, warranting further research.
